# Health Risks Associated With Benzene Exposure in Children: A Systematic Review

**DOI:** 10.1177/2333794X18789275

**Published:** 2018-08-17

**Authors:** Mark A. D’Andrea, G. Kesava Reddy

**Affiliations:** 1 University Cancer and Diagnostic Centers, Houston, TX, USA

**Keywords:** benzene poisoning, blood disorders, chemical exposure, health impact, hematological toxicity, hepatotoxicity, Illness symptoms, pediatric populations, psychological effects, respiratory function

## Abstract

Currently, there is a paucity of studies evaluating the adverse health effects of benzene exposure in children or clinical findings of those children who have been exposed. However, emerging studies show that benzene exposure can cause deleterious health effects in children. The objective of this study was to evaluate and summarize published studies on the adverse health effects of benzene exposure in children. More than 77 articles were examined and only the articles that dealt with adverse health effects on pediatric populations were included in the study. The evaluation of those studies provided current understanding of the health effects of benzene exposure in children. Findings from the currently available studies reveal that benzene exposure is associated with abnormalities in hematologic, hepatic, respiratory, and pulmonary functions in children. Published studies clearly support the need for further assessment of the potential adverse effects of benzene exposure in children, and clinical and laboratory findings of these children.

## Introduction

Benzene is a clear colorless flammable solvent with an almost sweet yet gasoline-like odor that easily volatilizes into vapors in air. It is a natural component of both crude and refined petroleum and is formed as a result of the incomplete combustion of fossil fuels such as petroleum products and coal.^1^ Benzene ranks in the top 20 most abundantly produced chemicals in the United States.^2^ It is a commercially important intermediate of many chemicals manufactured in the industry. In addition, benzene is the most widely used chemical in the synthesis of various polymers, resins, and synthetic fibers. More than 98% of the benzene produced is derived from the petrochemical and petroleum refining industries.^3^ The major sources of most of the ambient benzene is from petroleum refineries, emissions from coal and oil combustion, motor vehicle exhaust, evaporation from gasoline service stations, industrial solvents, and hazardous waste sites. Benzene is also a major component of tobacco smoke.^4^ As a volatile organic compound, it is one of the main contributors to air pollutants in the environment.^5,6^ It is found in the environment as a contaminant from both human activities and natural processes.^7,8^

Environmental benzene exposure is an important health concern. It has been clearly established that human exposure to benzene leads not only to hematologic cancers^9,10^ but also to a wide range of adverse noncancerous effects including functional aberration of respiratory, nervous, immune, hematological, hepatic, renal, cardiovascular, and reproductive systems.^5,11-15^ Additionally, benzene exposure can affect both B-cell and T-cell proliferation, reduce the host resistance to infection, and produce chromosomal aberrations.^16^ These deleterious health effects of benzene exposure have been very well established, especially in adults. However, there is a paucity of investigations evaluating the clinical findings and adverse health effects of benzene exposure in children. Although the literature on the health consequences of benzene in children is scant, emerging studies show that benzene exposure can cause deleterious health effects in children. Moreover, epidemiological evidence suggests that environmental benzene exposure is potentially a major cause of childhood leukemia and other hematologic cancers.^17-20^

Children at various developmental stages have unique physical risk factors when exposed to environmental toxins including benzene due to their levels of mobility, oxygen consumption, hormonal production, and overall growth. In addition, the toxicodynamic processes that determine exposure, absorption, metabolism, excretion, and tissue vulnerability are all age related.^21^ Moreover, children have a higher unit body weight exposure to benzene or other toxins than adults because of their heightened activity patterns and different ventilation tidal volumes and frequencies. Furthermore, children are more susceptible to leukemogenesis because their hematopoietic cell populations are differentiating and undergoing maturation. The incomplete metabolic systems, immature host defenses, high rates of infection by respiratory pathogens, and activity patterns make children more vulnerable to the toxic effects of benzene exposure.^22,23^ The physiology, immature enzyme systems, and clearance mechanisms play a critical role in determining the susceptibility of children to toxins.^21-24^ In particular, the pharmacokinetics of benzene differ widely between children and adults due to children’s incomplete metabolic systems, rapid tissue regeneration, immature host defenses, activity patterns, and high rates of infection by respiratory pathogens.^22,23^ Thus, children are more susceptible to the effects of environmental toxic pollutants. However, the susceptibility to benzene may vary due to its effect that arises, in part, from genetic variations in its metabolism, DNA repair, genomic stability, and immune function.

**Figure 1.**
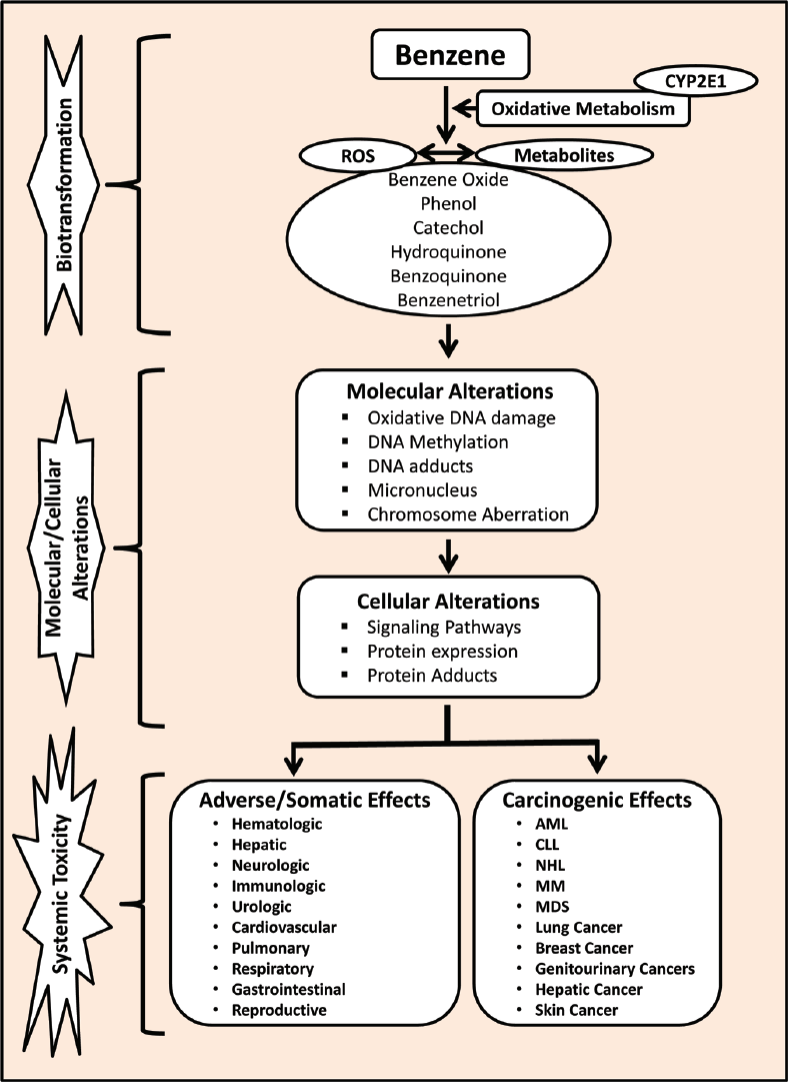
A schematic illustration of benzene metabolism, its mechanisms of toxicity, and its toxic effects in humans. Abbreviations: AML, acute myeloid leukemia; CLL, chronic lymphocytic leukemia; CYP2E1, cytochrome P450 2E1; MDS, myelodysplastic syndrome; MM, multiple myeloma; NHL, non-hodgkin lymphoma; ROS, reactive oxygen species.

The precise mechanism of benzene-induced toxicity is not completely understood but it is believed that there are multiple mechanisms of action involved in benzene toxicity (Figure 1).^25-27^ More specifically, the toxic effects of benzene are believed to arise from its metabolites such as benzene oxide, phenol, benzoquinone, muconaldehydes, hydroquinone, and catechol. Following absorption, benzene is metabolized by cytochrome P450 in the liver resulting in the production of its metabolites phenol, catechol, hydroquinone, and benzene oxide.^26^ These metabolites undergo further metabolism in the bone marrow to form a benzoquinone. Numerous studies have shown that many of these benzene metabolites are directly responsible for both its cytotoxic and genotoxic effects.^28-30^ In the bone marrow, formation of benzoquinone from the metabolism of benzene produces myelotoxicity due to its high reactivity to form adducts with proteins and DNA.^26,31^ These protein and DNA adducts interfere with the cellular functions and cause damage in the hematopoietic cells in addition to chromosomal aberration, oxidative stress, gene expression alteration, error-prone DNA repair, epigenetic regulation, apoptosis, and disruption of tumor surveillance.^32^ The generation of free radicals leading to oxidative stress, immune system dysfunction, and decreased immune surveillance has been described as the possible mechanisms underlying benzene-induced toxicity.^33^

Given the importance of the toxicity of benzene, this review article provides summaries of the current scientific knowledge and understanding of the clinical findings and health consequences of benzene exposure among children. Specifically, this article summarizes the quantitative changes in hematological and hepatic functions in addition to qualitative changes among somatic symptom in children exposed to benzene.

## Methods

We sought all published studies, primarily in the peer-reviewed literature using electronic databases such as MEDLINE via PubMed and Google Scholar. The combinations of the keyword “benzene exposure” with any of the association to the following terms was used for the search in the database search: children, pediatrics, adverse health effects, blood disorders, chemical exposure, hematological toxicity, hepatotoxicity, illness symptoms, psychological effects, and respiratory function. We also searched reference lists in those publications that we obtained in an attempt to find additional relevant publications. Nonindexed journals were manually searched. The search was restricted to English-language articles. Abstracts that had been published in English were also included in this study.

## Results

**Figure 2.**
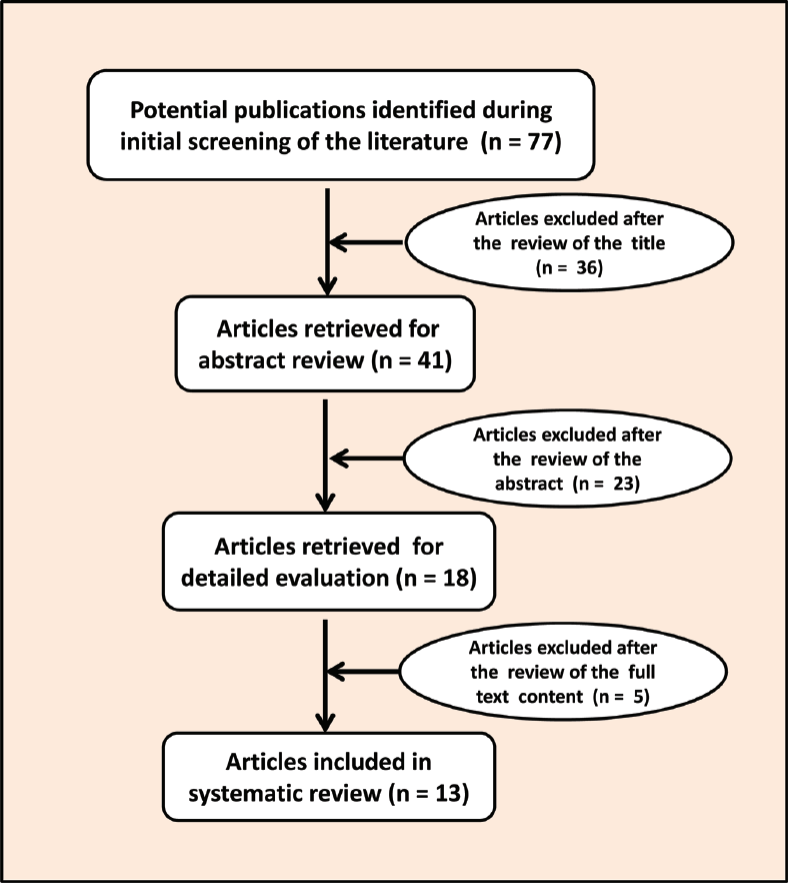
A flow chart illustrating the selection of articles for the study.

Figure 2 shows the steps involved in the selection process of the published articles for the study. On reviewing the articles’ titles, abstracts, and full text content of the study, most of the articles were excluded. The main reasons for exclusion were that they were either nonquantitative, nonanalytical, or lacked clinical data. Articles with clinical data were reviewed, and the information that related to the health effects of benzene exposure in children was assessed and summarized in this review article (Table 1).

**Table 1.** Summary of Studies on the Effect of Benzene Exposure Among Children.

Location of Study	Study Design	Children’s Age	Sample Size	Observed Clinical Health Effects	Reference
Ulsan, Korea	Cohort	8-11 years	192 (97 benzene exposed and 95 control) children	Reduced WBC, RBC, platelets, and lymphocytes counts, decreased hemoglobin in benzene-exposed children compared with unexposed children	Lee et al (2002)^34^
Texas City, TX	Cohort	8-11 years	312 (157 benzene exposed and 155 control) children	Reduced WBC counts, increased platelet counts, elevated creatinine levels, and increased liver enzymes such as ALP, AST, and ALT in benzene-exposed children compared with unexposed children	D’Andrea and Reddy (2013)^35^
Texas City, TX	Cohort	8-11 years	899 (641 benzene exposed and 258 control) children	Reduced WBC counts, increased platelet counts, decreased hemoglobin, hematocrit, and BUN levels, and increased liver enzymes such as ALP, AST, and ALT in benzene-exposed children compared with unexposed children	D’Andrea and Reddy (2016)^36^
Kanawha County, WV	Cohort	7-8 years	7796 children	Increased incidence of chronic respiratory symptoms in children attending schools located in a close proximity to chemical industries. Significant trends were observed for asthma-related responses such as a physician’s diagnosis of asthma, persistent wheezing, and attacks of shortness of breath with wheezing in school children enrolled within a close proximity to chemical plants regions than those in the nonindustrial region.	Ware et al (1993)^37^
La Plata, Argentina	Cohort	6-12 years	1 191 (282 living close to the petrochemical plants, 270 exposed to heavy traffic, and 639 living in nonpolluted areas)	Significantly elevated asthma and respiratory symptoms including wheezing, cough, dyspnea, and rhinitis, and reduced lung function in children living near the petrochemical plant compared with those living in nonpolluted areas	Wichmann et al (2009)^38^
Rio Grande do Norte, Brazil	Cross-sectional	0-14 years	209 children	Higher incidence of respiratory symptoms in children exposed to petrochemicals	Moraes et al (2010)^39^
El Paso, TX	Panel study	6-12 years	36 children	Increased Asthma Control Questionnaire score in children exposed to traffic pollution with benzene, toluene, and other toxins	Zora et al (2013)^40^
Asturias, Gipuzkoa, Sabadell, and Valencia, Spain	Cohort	12-18 months	2199 infants	Increased respiratory tract infections	Aguilera et al (2013)^41^
Los Angeles, CA	Panel study	10-16 years	21 children	Increased asthma and lung function among the children exposed to benzene	Delfino et al (2003)^42^
Viseu, Portugal	Panel study	6-8 years	51 children	Deteriorated lung function in children exposed to benzene	Martins et al (2012)^43^
Texas City, TX	Cohort	8-11 years	312 (157 benzene exposed and 155 control) children	Upper respiratory (67%), neurological symptoms (57%), diarrhea (25%), cough (24%), dermatological (24%), nausea/vomiting (21%), gastrointestinal (12%), wheezing (9%), chest pain (6%), vision (6%), painful joints (6%), and urinary irritation (3%)	D’Andrea and Reddy (2016)^44^

Abbreviations: WBC, white blood cells; RBC, red blood cells; ALP, alkaline phosphatase; AST, aspartate aminotransferase; ALT, alanine aminotransferase; BUN, blood urea nitrogen.

### Hematological Effects of the Benzene Exposure in Children

A cohort study by Lee and coauthors^34^ assessed the hematological changes in children living near the petrochemical estate region in Ulsan, Korea, who were environmentally exposed to volatile organic compounds containing low levels of benzene. This study included a total of 192 children between the ages of 8 and 11 years who were living in close proximity to a petrochemical estate region or suburban region of Ulsan, Korea. The exposed group was composed of 48 boys and 49 girls who lived near the petrochemical estate region and went to an elementary school located near the petrochemical estate. The unexposed group was composed of 46 boys and 49 girls who had lived in the suburban region 10 miles from the petrochemical estate region. Both unexposed and benzene-exposed groups had similar age and sex distributions. Hematological assessment revealed that the total white blood cell (WBC) counts and absolute lymphocytes counts of 11-year-old children living near the petrochemical estate region were significantly lower than those of children living in the suburban region (*P* = .009, *P* = .032, respectively). Although the 8-year-old children living near the petrochemical estate region had decreased WBC counts and absolute lymphocytes counts compared with those living in the suburban region, they did not reach statistical significance. The red blood cell (RBC) counts and hemoglobin levels of the 8-year-old exposed children were significantly lower than those of the unexposed children (*P* < .001, *P* < .001, respectively). A similar, but not statistically significant, trend was seen in the parameters in the 11-year-old exposed and unexposed groups. Whereas the platelet counts were significantly decreased in both 8- and 11-year-old exposed children compared with unexposed children (*P =* −.001, *P =* −.001, respectively). A follow-up assessment at 3 and 6 months after the initial evaluation yielded similar differences but there were not consistent findings in the exposed and unexposed groups of the 8- and 11-year-old children.

The generalized linear model analysis of variance for the complete blood count values showed that the region where the exposure took place was a significant independent variable for the total WBC counts, RBC counts, and platelet counts (*P* = .007, *P* = .004, and *P* = .036, respectively), and the children’s sex was a significant independent variable for the RBC counts (*P* = .001). Similarly, age was a significant independent variable for the total WBC counts, absolute lymphocyte counts, and platelet counts (*P* < .001, *P* = .004, and *P* = .005, respectively). Overall, the study findings showed that environmental exposure to volatile organic compounds containing low levels of benzene was associated clinically with a higher prevalence of hematological abnormalities in children living near the petrochemical estate region.

A pilot study by D’Andrea and Reddy^35^ evaluated the hematological function in children who were less than 17 years old and exposed to benzene following British Petroleum’s (BP) flaring incident in Texas City, Texas. A total of 312 children were included in the study. Of the 312 children, 157 were exposed to benzene and 155 were not exposed to benzene. Both unexposed and benzene-exposed groups had similar age and sex distributions. Clinically, hematologic analysis showed that WBC counts were significantly decreased in benzene-exposed children compared with the unexposed children (*P* = .022). Conversely, the platelet counts were increased significantly in the benzene-exposed group compared with the unexposed group (*P* = .005). Similarly, the serum creatinine levels were significantly increased in the benzene-exposed children compared with the unexposed children (*P* = .000). However, no significant alterations were observed in the mean hemoglobin or hematocrit or blood urea nitrogen levels between the benzene exposed and unexposed children. The results of this pilot study indicated that environmental exposure to benzene is associated clinically with altered hematological profiles in those children who were exposed to the benzene from the flaring incident at the BP refinery facility in Texas City, Texas.

A later larger cohort study by the same authors assessed the hematological changes in children exposed to benzene following the flaring incident.^36^ A total of 899 children aged <17 years were included in the study. Of the 899 children, 258 were unexposed and 641 were exposed to benzene. The mean age of the unexposed and exposed children was 10.5 and 9.5 years, respectively. Among the unexposed children, there were 57% male and 43% female children. In the benzene-exposed group, there were 52% males and 48% females.

Hematological analysis indicated that those children exposed to benzene had significantly decreased mean WBC counts compared with the unexposed children (*P* = .001). Conversely, the mean platelet counts in the benzene-exposed group were significantly higher when compared with the unexposed children group (*P* = .001). Whereas the mean hemoglobin levels decreased significantly in the benzene-exposed group compared with the unexposed group (*P* = .001). Similarly, the percentage of hematocrit decreased significantly among the benzene-exposed children compared with the unexposed children (*P* = .001). Blood urea nitrogen was also found to be reduced significantly in benzene-exposed group compared with the unexposed group (*P* = .001). However, no significant differences were noted in the serum creatinine levels between the benzene exposed and unexposed groups. Furthermore, subanalysis indicated that, regardless of age or gender, significant alterations in the hematological profiles were seen in those children exposed to benzene. Overall, the findings of the hematological profiles confirmed the pilot study findings indicating that children who have been exposed to benzene have significantly increased health risks compared with unexposed children.

### Effect of Benzene Exposure on Hepatic Function in Children

Currently, there are no published studies in literature that evaluated the clinical effect of benzene exposure on the liver function in children except 2 recent reports published by the authors.^35,36^ The initial pilot study included 157 benzene-exposed and 155 unexposed children and assessed their liver function enzymes such as alkaline phosphatase (ALP), aspartate aminotransferase (AST), and alanine aminotransferase (ALT). The study findings revealed that benzene-exposed children had clinically significantly higher levels of ALP (*P* = .04), AST (*P* = .015), and ALT (*P* = .005) compared with the unexposed children.

Subsequently, the larger cohort study^36^ assessed the liver function enzymes in 641 benzene-exposed children and compared with the 258 unexposed children. Serum ALP, AST, and ALT levels were reported to be increased significantly in children exposed to benzene compared with the unexposed children (*P* = .001). Furthermore, subgroup analysis indicated that, regardless of age or gender, significant alterations in hepatic enzymes were seen in children exposed to benzene. Overall, the findings of the hepatic profiles confirmed the pilot study findings indicating that children who have been exposed to benzene have significantly increased health risks compared with unexposed children.

### Benzene Exposure and Illness Symptom Profiles in Children

Among all, respiratory illness symptoms are the most often studied health complaints in children exposed to benzene or petrochemicals/urban traffic pollutants. Upper respiratory symptoms were the most (67%) frequently reported, followed by neurological symptoms (57%), diarrhea (25%), and cough (24%). Logistic regression analysis indicated that neurological symptoms (*R*^2^ = 0.75), chest pain (*R*^2^ = 0.64), joint pain (*R*^2^ = 0.57), and vision difficulty (*R*^2^ = 0.54) were positively associated with increasing age. Other studies have shown that asthma symptoms such as those related to wheezing, cough, and shortness of breath or chest tightness were the most frequently reported respiratory illness symptoms in benzene-exposed children. A study by Ware and co-investigators^37^ evaluated respiratory and irritant health effects of ambient volatile organic compounds in 7796 children attending 74 elementary schools located in chemical industry regions. The findings indicated that exposure to volatile organic compounds from chemical manufacturing plants were associated with an increased incidence of chronic respiratory symptoms in children attending schools located in a close proximity to chemical industries. Significant trends were observed for asthma-related responses such as a physician’s diagnosis of asthma, persistent wheezing, and attacks of shortness of breath with wheezing in school children enrolled within a close proximity to regions containing chemical plants than those in the nonindustrial regions.

Similar findings were reported in a study by Wichmann et al^38^ that assessed the effects of exposure to petrochemical pollution on the respiratory health of children aged 6 to 12 years living close to petrochemical plants (n = 282) and compared them with those living in a region with exposure to heavy traffic (n = 270) or in relatively nonpolluted areas (n = 639) in La Plata, Argentina. The findings showed that children living near the petrochemical plant had significantly elevated asthma and respiratory symptoms (wheezing, cough, dyspnea, and rhinitis) and significantly reduced lung functions than those living in nonpolluted regions (*P* < .001). Moraes and coworkers^39^ investigated the health impacts of living near petrochemical plants by assessing respiratory illnesses in 209 Brazilian children. The results from this study revealed that respiratory symptoms were found to be increased in children among communities in the vicinity of a petrochemical complex particularly those living downwind from the plant.

A panel study conducted by Zora et al^40^ assessed the associations between urban air pollution of benzene and pediatric asthma control using an Asthma Control Questionnaire (ACQ) score in 2 elementary schools located in high- and low-traffic areas of El Paso, Texas. Eligibility criteria included age of the children between 6 and 12 years, a physician diagnosis of asthma, no other lung disease or major illness, a nonsmoking household, and residence proximal to their school. Data were reported for 36 of the 38 children who completed the protocol. The study found that benzene levels in the air of a school located in the high-traffic area ranged from 0.2 to 2.4 µg/m^3^. Although no significant associations between benzene and other pollutants with an increase in ACQ score were found, an increase in ACQ score was related with an increase in benzene levels among children inhaling corticosteroids daily. Aguilera et al^41^ investigated the association of air pollution exposure during pregnancy and respiratory illnesses, ear infections, and eczema during the first 12 to 18 months of life in a Spanish birth cohort of 2199 infants. These authors observed that during the second trimester of pregnancy, an increase in 1.0 µg/m^3^ of benzene exposure was associated with an increased risk of lower respiratory tract infections in those infants.

In a panel study, Delfino et al^42^ examined the longitudinal relationship of the daily asthma severity among asthmatic children exposed to volatile organic compounds such as benzene. The study included 21 asthmatic children between 10 and 16 years of age. The study revealed that increased mean concentrations of benzene (5.7 µg/m^3^) levels were associated with increased asthma and poor lung function among the children. Martins and coauthors^43^ evaluated the relationship between air polluted by benzene exposure and airway changes in a group of wheezing children. The investigators included a total of 51 wheezing children with a mean age of 7.3 years from Viseu, Portugal. Benzene levels were monitored for 4 weeks, and using a dispersion model, personal exposure was determined based on time-activity patterns according to the estimations. These authors reported that an increase in 10.0 µg/m^3^ of benzene exposure was associated with deteriorated lung function-related outcomes in wheezing children.

In a pilot study, we investigated the clinical presentation of the illness symptoms experienced by children who were exposed to benzene following a flaring incident at the BP refinery in Texas City, Texas.^35^ The study included a total of 157 children who were exposed to benzene. Among the illness symptoms, neurological symptoms such as unsteady gait, memory loss, and headaches were the most (80%) frequently reported symptoms in children exposed to benzene. Upper respiratory symptoms were reported by 48% of the benzene-exposed children followed by cough (48%), nausea/vomiting (43%), dermatological (36%), shortness of breath (32%), wheezing (27%), dizziness (22%), chest pain (15%), painful joints (15%), and weight loss (13%). To complement these findings, recently we conducted a full-fledged study in 641 children who were exposed to benzene following a flaring incident at the BP refinery in Texas City, Texas.^44^ A total of 1790 illness symptoms were observed in 641 children exposed to benzene.

Among all clinically presented illness symptoms, upper respiratory symptoms occurred as the most frequently (67%) followed by neurological symptoms (57%), diarrhea (25%), and cough (24%). Logistic regression analysis indicated that neurological symptoms (*R*^2^ = 0.75), chest pain (*R*^2^ = 0.64), joint pain (*R*^2^ = 0.57), and vision difficulty (*R*^2^ = 0.54) were positively associated with increasing age of the children. Overall, the findings revealed that children exposed to benzene experienced range of illness symptoms indicating their vulnerability to increased risks and health complications.

## Discussion

The literature reviewed in this article indicates there is a growing interest in evaluating the clinical and health consequences of benzene exposure among children. The literature on both clinical and health effects of benzene exposure in children is scarce, and studies evaluating the hematological, hepatic, and respiratory effects of benzene exposure are starting to emerge based on established biological mechanisms of benzene toxicity. Overview of the findings of the studies included in this review indicates that benzene exposure among children was clinically associated with alterations in hematologic, hepatic, and respiratory functions. In addition, benzene exposure was associated with the clinical presentation of several illness symptoms in children.

Clinical evidence further suggests that hemotoxicity is the major effect and is unique to benzene. Exposure to benzene causes bone marrow injury resulting in hemotoxicity leading to changes in WBCs, platelets, hemoglobin, hematocrit, and other blood cells formation. Multiple mechanisms including alterations in the expression of numerous genes and proteins, DNA methylation patterns, and RNA profiles appear to play an important role in benzene-induced hemotoxicity in exposed children.^27^

Although several studies have investigated the effect of benzene exposure on the hematological changes in adults, only a handful of studies published so far have evaluated the clinical changes in the hematological functions among children following their exposure to benzene.^34-36^ The findings of these studies demonstrate that children exposed to benzene experienced significantly reduced hematological indices compared with those unexposed children. However, conflicting findings in platelet counts were observed in benzene-exposed children. Our recently published studies demonstrated significantly elevated platelet counts in children who were exposed to benzene compared with unexposed children.^35,36^ However, in the study reported by Lee and associates,^34^ significantly decreased platelet counts were observed in children exposed to benzene compared with unexposed children. Although the discrepancies in the platelet counts in benzene-exposed children currently cannot be explained, Ceresa and coworkers^45^ previously found that thrombocytopenia was not a constant finding in most of the adult subjects who were exposed to benzene. Nevertheless, additional studies are warranted to clarify the effect of benzene exposure on the platelet counts in children.

The liver is the principal organ of xenobiotic metabolism, and hence, it is very important to monitor its function in people exposed to benzene or other toxins. It is well known that phosphatases, aminotransferases, and dehydrogenases are important enzymes in the biological processes. They are involved in the detoxification, metabolism, and biosynthesis of energetic macromolecules for different essential functions. Any interference in these enzymes leads to biochemical impairment and changes in the tissue and cellular function. Thus, the measurement of these liver enzyme such as ALP, AST, and ALT are routinely assessed as indicators for hepatic dysfunction and damage.^46,47^ In normal conditions, these enzymes are confined to the cells but are released into circulating blood when there is necrosis or injury. Despite its importance, until recently, there were no published studies available in the literature evaluating the effect of benzene exposure on the hepatic function in children. The 2 recent studies reported by the authors^35,36^ revealed that the serum levels of ALP, AST, and ALT were found to be elevated among those children who were exposed to benzene indicating hepatic abnormalities in these children. The increase in the levels of these liver enzymes in their serum suggests the impairment of the hepatic function in children exposed to benzene.

Studies assessing the somatic or clinically presenting illness symptoms such as respiratory, neurological, gastrointestinal, and other symptoms in children exposed to benzene were also limited in the published literature. However, evidence from available studies suggests that benzene exposure is associated clinically with sickness symptoms in children. The most common clinical presentations of the illness symptoms include neurological, respiratory, shortness of breath, wheezing, dizziness, chest pain, and painful joints.

## Conclusions

Together, studies evaluating the clinical changes in the hematologic, cardiac, hepatic, renal, and other vital organ functions in children who were exposed to benzene are sparse. We have yet to learn and understand the full extent of all the adverse effects that benzene exposure has on pediatric populations. Findings from the currently available studies reveal that benzene exposure is associated with clinical abnormalities in the hematologic, hepatic, respiratory, and pulmonary functions in children. The hematological abnormalities were characterized by changes in RBC, WBC, absolute lymphocytes, platelets, hemoglobin, hematocrit, and creatinine in benzene-exposed children. Similarly, the hepatic abnormalities were characterized by elevated levels of ALP, AST, and ALT enzymes in the serum of the children exposed to benzene. Few studies have evaluated the somatic or illness symptoms such as respiratory, neurological, gastrointestinal, and other symptoms in children exposed to benzene. These findings indicate that exposure to benzene may lead to clinically detectable detrimental health effects in children. However, to fully understand the importance and nature of these effects, further longitudinal and mechanistic studies on the health effects of benzene exposure in children are warranted.
